# Natural Killer Cell-Dependent Anti-Fibrotic Pathway in Liver Injury via Toll-Like Receptor-9

**DOI:** 10.1371/journal.pone.0082571

**Published:** 2013-12-10

**Authors:** Lina Abu-Tair, Jonathan H. Axelrod, Sarit Doron, Yossi Ovadya, Valery Krizhanovsky, Eithan Galun, Johnny Amer, Rifaat Safadi

**Affiliations:** 1 Liver & Gastroenterology Units; Division of Medicine, Hadassah Medical Center, Jerusalem, Israel; 2 Goldyne Savad Institute of Gene Therapy, Hadassah Medical Center; Jerusalem; 3 Department of Molecular Cell Biology, Weizmann Institute of Science, Rehovot, Israel; University of Nebraska Medical center, United States of America

## Abstract

The toll-like receptor-9 (TLR9) agonist cytosine phosphate guanine (CpG), activates hepatic stellate cells (HSCs) and mediates fibrosis. We investigated the TLR9 effects on lymphocyte/HSCs interactions. Liver fibrosis was induced in wild-type (WT) mice by intra-peritoneal carbon-tetrachloride (CCl_4_) induction for 6 weeks. Fibrotic groups were intravenously treated by a vehicle versus CpG along last 2 weeks. Compared to vehicle-treated fibrotic WT, the *in-vivo* CpG-treatment significantly attenuated hepatic fibrosis and inflammation, associated with decreased CD8 and increased NK liver cells. *In-vitro*, co-cultures with vehicle-treated fibrotic NK cells increased HSCs proliferation (P<0.001) while their CpG-treated counterparts achieved a significant decrease. To investigate the role of lymphocytes, TLR9^-/-^ mice induced-hepatic fibrosis were used. Although TLR9^-/-^ mice manifested lower fibrotic profile as compared to their wild-type (WT) counterparts, senescence (SA-β-Gal activity) in the liver and ALT serum levels were significantly greater. In an adoptive transfer model; irradiated WT and TLR9^-/-^ recipients were reconstituted with naïve WT or TLR9^-/-^ lymphocytes. The adoptive transfer of TLR9^-/-^ versus WT lymphocytes led to increased fibrosis of WT recipients. TLR9^-/-^ fibrotic recipients reconstituted with TLR9^-/-^ or WT lymphocytes showed no changes in hepatic fibrosis severity or ALT serum levels. TLR9 activation had inconsistent effects on lymphocytes and HSCs. The net balance of TLR9 activation in WT, displayed significant anti-fibrotic activity, accompanied by CD8 suppression and increased NK-cells, activity and adherence to HSCs. The pro-fibrotic and pro-inflammatory properties of TLR9^-/-^ lymphocytes fail to activate HSCs with an early senescence in TLR9^-/-^ mice.

## Introduction

Toll-like receptors (TLRs) are part of the innate immune system [1], members of which are expressed by hepatic stellate cells (HSCs) [2]. TLR4 activation has been shown to augment HSCs activation and hepatic fibrosis [3]. Furthermore, TLR3 recognizes double stranded RNA, such as polyinosinicpolycytidylic acid (poly I: C), to induce a potent innate immune response that includes the production of interferon type I and type II. NK cell activation by TLR-3-DCs depends on secretion of IL-12p70 [4].

TLR9, a member of the TLR family, is expressed intracellularly, within endosomal compartments [5], and activates innate immune defenses against viral and bacterial infection upon binding to DNA rich in cytosine phosphate guanine (CpG) motifs [6]. TLR9 expressed by HSCs is required for HSCs activation by both TLR9 agonists (CpG) and apoptotic hepatocyte DNA, and is thought to contribute to the development of liver fibrosis [7]. Watanabe et al. has demonstrated that host-derived denatured DNA from apoptotic hepatocytes induces a differentiation of HSCs via TLR9 through up-regulation of TGF-β1 and Collagen-1 in HSCs. Apoptotic hepatocyte DNA can increase the amount of matrix deposition and induce morphological changes known to be associated with HSC activation via TLR9. The relative inability of DNA from healthy hepatocytes to induce these changes further supports that this is an apoptosis-dependent phenomenon, and is therefore a candidate mechanism by which in vivo hepatocyte apoptosis results in HSC differentiation and fibrosis [8]. Consistent with this observation, TLR9 deficiency in mice ameliorates the development of fibrosis following liver injury induction [9]. 

The TLR9 activation was reported to mediate the stimulation of natural killer (NK) cells, observed both through *in vitro* and *in vivo* settings [10,11,12], the therapeutic potential of TLR9 agonist was even suggested [13]. Hepatic NK cells, on the other hand, display an anti-fibrotic activity through a direct killing of the activated HSCs [14,15,16]. Thus, we hypothesize that TLR9 triggering would generate an NK-mediated immune anti-fibrotic response.

In the current study, direct HSCs *in vitro* stimulation by CpG (TLR9 agonist) was reproduced [8]. However, CpG administration in wild-type (WT) mice modulated liver lymphocytes into an anti-fibrotic pattern, which was confirmed by *in vitro* co-cultures with HSCs. In TLR9 knockout (TLR9^-/-^) liver injury, pro-fibrogenic TLR9^-/-^ lymphocytes fail to stimulate senescent TLR9^-/-^HSCs. However, in the adoptive transfer model they increased liver injury and HSCs activation of WT recipients. 

## Materials and Methods

### Animals and Hepatic-Fibrosis Model

Twelve-week C57BL/6 old-male wild-type (WT) and Toll-like receptor 9 Knockout (TLR9^-/-^) mice [17,18] were used. This study was carried out in strict accordance with the recommendations in the guide for the care and use of laboratory animals of the National Institutes of Health. The protocol was approved by the local committee on the ethics of animal experiments of the Hebrew University (Permit Number: MD-10-12516-2). Hepatic fibrosis was induced in WT and TLR9^-/-^ animals using carbon tetrachloride (CCl_4_). CCl_4_ was diluted to 10% in corn oil and introduced by intra-peritoneal injections at dosages of 0.5 µl pure CCl_4_/g body weight twice a week, for 6 weeks. There were 16 animals included in each presented study group. Animals receiving corn oil vehicle were used as controls. All surgery was performed under sodium pentobarbital anesthesia, and all efforts were made to minimize suffering. When sacrificed, and serum, livers and cells were harvested 3 days after the final dose of CCl_4_.

### TLR9 Activation by Oligodeoxynucleotides (ODNs) with Immunomodulatory (CpG) Motifs

During the fifth and sixth weeks of CCl_4_ injections in WT experiments, animals were treated once a week by hydrodynamic-based *in vivo* transfection, to ensure efficient and prolonged liver-directed therapeutic effects [19] with 20μg CpG ODN 1826 (Cat. code tlrl-1826 . 5'-TCC ATG ACG TTC CTG ACGTT-3’) or non- CpG ODN 1826 control (as a vehicle) (Cat. code tlrl-1826c 5’- TCC ATG AGC TTC CTG AGC TT-3’ (20 mer, purchased from Invivogen, Toulouse, France). 

### Total Body Irradiation Model

Mice were sub-lethally irradiated with a single total-body dose of 700cGy from a dual Cs-source (dose rate of 62cGy/min) to suppress lymphocyte-proliferation [20]. 

### The Adoptive Transfer Model

Lymphocytes were isolated from the spleens of WT and TLR9^-/-^ naïve donors. Million cells from each strain were repeatedly replenished by intra- peritoneal injections once per week for 4 weeks, either to irradiated WT or TLR9^-/-^ recipients, respectively. In the course of these 4 weeks, recipients were induced either with or without fibrosis by the CCl_4_ regimen [21]. 

### Serum Alanine Aminotransferase (ALT) and Cytokine Assessments

Serum ALT levels were measured by the automated enzymatic assay with the Vistros Chemistry Systems 950. To measure serum interferon gamma (IFNγ), and IL-10 levels, OptEIA ELISA kits (Pharmingen, San Diego, CA) were used according to the manufacturer's protocol [22]. 

### Western Blot Analysis

Liver samples were immediately frozen at -70°C for western blot analysis of liver protein extracts. The α-Smooth Muscle Actin (αSMA) was used as a marker of HSCs activation and hepatic fibrosis severity and β-actin as a housekeeping gene. Densities of immunoblot bands were measured and the αSMA/β-actin ratios were calculated as previously described [23]. 

### Hepatic hydroxyproline contents

Liver homogenates (100 µl each) were used, then add to 100 µl of concentrated HCl (12N), into a glass vial screwed up with teflon cap and hydrolyzed at 120°C for 8 h. hydrolyzed samples were transferred to a microcentrifuge tube and spinned at 10,000 rpm for 3 minutes to remove hydrolyzed residues from the sample, supernatants were used for the assay. Briefly, according to manufacturer's instructions (© Chondrex, Inc. 2607 151st Place NE, Redmond, WA), samples were distributed to a 96-well ELISA plate, Incubated with chloramine-T solution at R.T for 20 minutes, Then incubated with DMAB (dimethylaminobenzaldehyde) solution for 30 minutes at 60°C . Absorbance was measured at 550 nm. Hydroxyproline levels were calculated then converted into collagen level by the following equation: Hyrdoxyproline level (µg/ml) x 100/ 13.5= Collagen level (µg/ml). "

### Histological Assessment of Liver Injury

Sections fixed with 10% formalin were stained in 0.1% Sirius Red F3B in saturated picric acid (Sigma). The extent of fibrosis in Sirius Red-stained slides was evaluated using the Bioquant computerized morphometric method [23]. 

### Staining for Senescence-Associated β-Galactosidase Activity

Staining of SA-β-gal was performed at pH=5.5 for mouse tissue and pH=6.0 for human cells. Frozen sections of liver tissue, or adherent cells were fixed with 0.5% Gluteraldehyde in PBS for 15 min, washed with PBS supplemented with 1mM MgCl_2_ and stained for 5–6hrs in PBS containing 1 mM MgCl_2_, 1mg/ml X-Gal and 5 mM of each Potassium ferricyanide and Potassium ferrocyanide. Sections were counterstained with Eosin [15,24,25].

### Liver Lymphocyte and NK Isolation

Intra-hepatic lymphocytes were isolated by perfusion of the liver with digestion buffer. After perfusion, the liver was homogenized and incubated at 37 °C for 30 min. The digested liver cell suspension was centrifuged to remove hepatocytes and cell clumps. The supernatant was then centrifuged to obtain a pellet of cells depleted of hepatocytes to a final volume of 1 ml. Lymphocytes were then isolated from this cell suspension using 24% metrizamide gradient separation [23]. Cells were cultured or counted and stained for FACS analysis. For spleen cells; the spleens were meshed through cell strainers (40µm BD FALCON, USA), cells were sedimented by centrifugation at 800xg for 3 minutes, and then treated with lysis buffer at RT for 3 minutes, 9mL DMEM added and spinned as before [26]. Liver NK from lymphocytes were further isolated using a magnetic cell sorting kit (Miltenyi Biotec) according to manufacturer’s instructions.

### Primary Hepatic Stellate Cell Isolation

HSCs were isolated from male C57BL/6 mice. Livers underwent perfusion via the heart apex with 5-10 mL Gey's balanced salt solution (GBSS; Gibco BRL, Rockville, MD). Six donor livers were pooled for Pronase and Collagenase digestion at 37°C for 40 minutes in 20 mL of GBSS solution containing 0.1% (wt/vol) Pronase, 0.1% (wt/vol) Collagenase and 0.01% (wt/vol) deoxyribonuclease (DNase I). All enzymes were purchased from Roche Diagnostics GmbH, Mannheim, Germany. The resulting suspension was filtered through a 150 mm steel mesh and centrifuged on 17.5% Nycodenz cushion (Sigma, St. Louis, MO USA) at 1,400g for 20 minutes at 25°C, which produced an HSC-enriched fraction in the upper whitish layer. Cells were washed by centrifugation (400*g*, 25°C, 10 minutes) and cultured in Dulbecco's modified Eagle's medium supplemented with 10% (vol/vol,) fetal calf serum, 100 μg/mL penicillin, and 100 μg/mL streptomycin, for confluence. 

### Co-culture of Murine Primary HSCs with NK cells

HSCs were cultured in 10% DMEM at a density of (10^5^cells/ well) on a 6-well plate, with direct CpG incubation *versus* medium vehicle, or with liver NK cells (10^6^/well) that were isolated from the earlier WT experiment: naive and fibrotic donors that were treated or not (*in vivo*) with 20 μg/mouse CpG. After 48 co-culture hours, floating cells were washed and adhered cells were harvested and analyzed by flow cytometry as described below.

### Flow Cytometric Analysis

Liver lymphocytes (*in vivo*) and harvested adhered HSCs/ lymphocytes (following *in vitro* co-culture) were adjusted to 10^6^/ml in staining buffer (saline containing 1% bovine albumin; Biological Industries, Israel). Liver lymphocytes were stained for CD45 as a pan-leukocyte marker, for CD4, CD8 and NK1.1 as a NK marker. All flourochrome antibodies were purchased from eBioscience, Inc. Harvested HSCs/ NK cells were stained for CD45 and NK1.1. Then, stained cells were fixed with 4% paraformaldehyde for 10 minutes and permeabilized with 0.1% saponine in Phosphate buffered saline for 20 minutes and then stained with anti-human αSMA-PE (phycoerythrin)-conjugated monoclonal antibody (R&D Systems USA).

Stained cells were analyzed with a flow cytometer (LSR II bdbiosciences.com, BD FACSDiva software version 6.0). The CD45 positive population was gated in the case of freshly isolated liver lymphocytes. CD4, CD8 and NK cells are presented as percentage of the CD45 population. Cultured cells with double positive NK1.1 and αSMA were considered as the adhered cells. NK cells were also assessed for the frequency of degranulation by the CD107a staining [26]. 

### Proliferation Assay

Cultured HSCs were washed following harvesting and stained with CFSE (5,6-carboxyfluorescein diacetate) according to the manufacturer’s protocol (Invitrogen, Oregon). Briefly, CFSE staining of HSCs after labeling is extremely high fluorescence. The majority of CFSE initially taken up by the cells is lost within the first few days following proliferation. The more the decrease in fluorescence the higher the cells proliferate. The units obtained are the Mean fluorescence intensity (MFI- arbitrary unit). Results were calculated as the fold decrease in MFI in day 5 as compared to day Zero and analyzed by flow cytometry.

### Statistical Analysis

Results are presented as mean values ± standard deviation. Standard-error is used for the Bioquant® analysis. Student’s t-Test and ANOVA are used for statistical significant correlations. *P* value <.05 was considered significant.

## Results

### CpG (ODNs) Treatments Attenuate Hepatic Fibrosis and Inflammation in WT animal model

**Figure 1:**
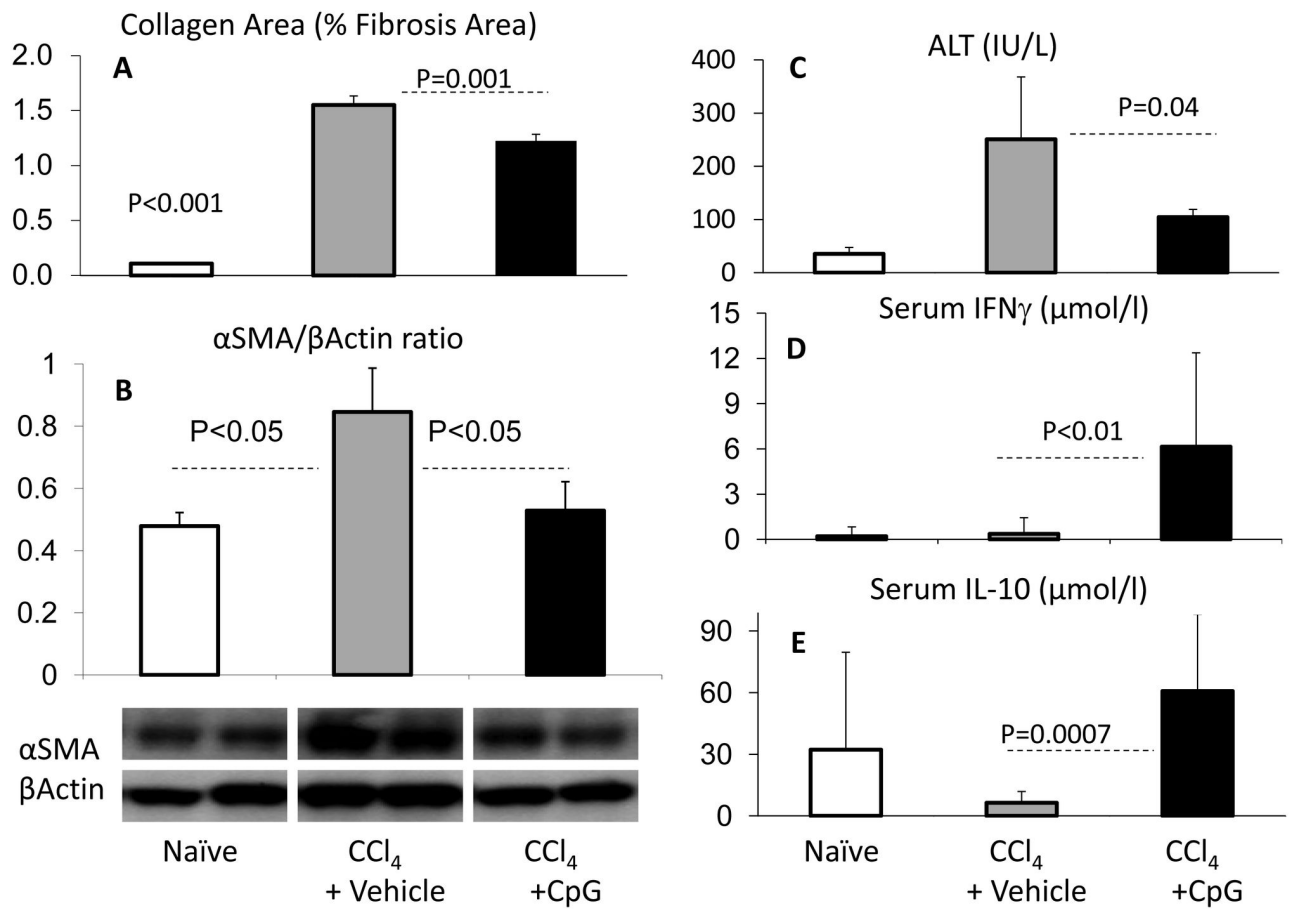
*In* *vivo* effects of treatment with CpG on chronic liver cell injury. WT CCl_4_-fibrotic mice were treated with CpG motifs (black bars) versus vehicle injections (gray bars) and compared to naïve animals (plain bars). A) Liver fibrosis extent was evaluated by Sirius-red-stain; quantified by Bioquant® computerized method and represented as arbitrary units of mean relative collagen (fibrosis) area ± standard error, B) and by calculated densitometry of western blotting bands as αSMA/β-actin ratio. CpG-treated mice showed significantly less collagen area and αSMA/β-actin ratio compared to WT vehicle-treated fibrotic groups. C) ALT serum levels followed the same fibrosis pattern. D) Serum levels of cytokines from the CpG-treated fibrotic animals had a significant increase of IFNγ and E) IL-10 levels; as compared to vehicle treated fibrotic controls.

To study the effect of TLR9 activation on hepatic fibrosis model; CpG (TLR9 agonists, 20 µg/mouse) were administered by a hydrodynamic transfection manner into fibrotic mice model receiving the CCl_4_ injections as described in materials and methods. Mice groups receiving either the non-CpG ODNs or/and the corn oil were used as vehicle controls. Along all the experiments with the hepatic fibrosis model, mice receiving the corn oil (vehicle-treated) had consistent results with the WT naïve mice (data not shown). The isolated livers were histologically evaluated for fibrosis extent by Sirius-red-staining using the Bioquant® computerized quantitations method and by αSMA liver protein expression. Both fibrotic mice receiving either the vehicle or the CpG-ODNs showed a significant elevation in their relative collagen (fibrosis) area (p<0.001) as compared to naïve non-fibrotic mice. However, the CpG-treatment showed less collagen area of 1.22 ± 0.06 (Figure 1A) as compared to vehicle-treated controls (1.55 ±0.08; *P*=0.001). Liver protein extracts in the CpG group also showed a significant decrease in αSMA expressions by western blot analysis (Figure 1B). The calculated αSMA/β-actin ratio of densitometry showed consistent patterns, upper part of (Figure 1B). 

Fibrotic mice receiving the CpG showed lower serum ALT levels of 104.40± 14.5 IU/L as compared to 251.75± 117.2 IU/L in control vehicle treated group (*P*=0.05), which further indicates amelioration of liver injury (Figure 1C). 

In parallel, serum IFNγ levels (Figure 1D) as well as serum IL-10 levels (Figure 1E) increased significantly in the CpG-treated fibrotic animals (*P*<0.005) as compared to vehicle-treated fibrotic controls, suggesting an anti-fibrotic as well as an anti-inflammatory phenotypic change [27]. 

### Anti-Fibrotic Pattern of Intra-Hepatic Lymphocyte Subsets

**Figure 2:**
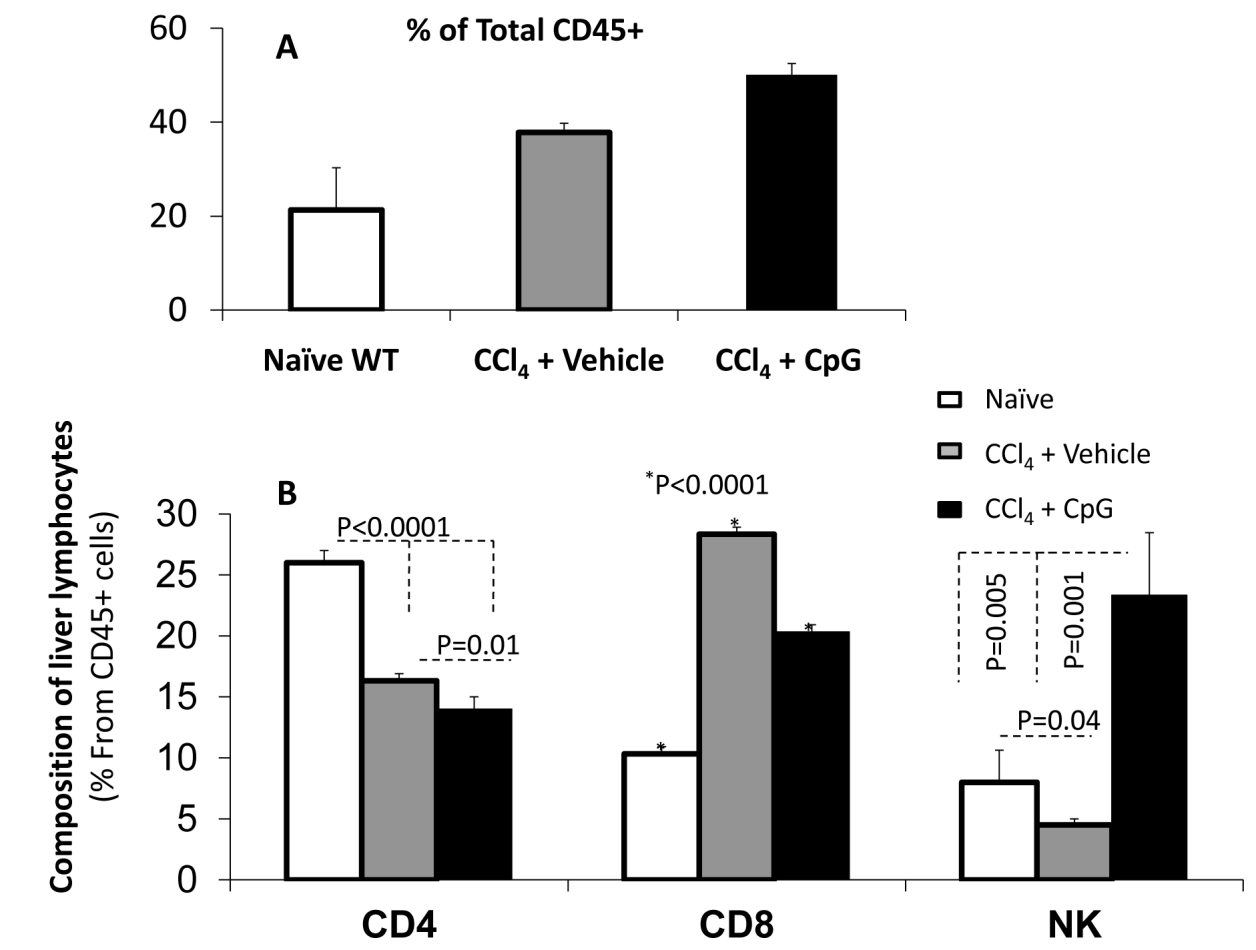
Flow cytometry analysis of isolated intra-hepatic lymphocytes. A) Total percent of CD45 from livers of fibrotic animals showed increased infiltrate in the CpG groups as compared to the vehicle. B) A significant augmentation of liver CD8 content; along with CD4 and NK reductions in vehicle-treated CCl_4_ fibrosis as compared to naive animals. The CpG therapy significantly decreased the CD4, and CD8 populations, but markedly increased the NK population up to 3-fold of expression.

Polymorphonuclear cells including intra-hepatic lymphocytes were isolated and identified for the anti-CD45 (pan-leukocyte marker) (Figure 2A) using the flow cytometry analysis. Total percent of CD45 from livers of the Fibrotic mice animal groups showed increased infiltrates in liver of mice receiving the CpG motifs as compared to their counterparts with the vehicle treatment. Furthermore, the intra-hepatic lymphocyte content (Figure 2B) revealed a significant augmentation of liver CD8 content (*P*<0.0001), a reduction in CD4 (*P*<0.0001) and NK (*P*=0.04) levels in vehicle-treated CCl_4_ fibrosis-induced animals. CpG treatment significantly decreased the liver CD4 (*P*=0.01), and CD8 (*P*<0.0001) populations, but markedly increased the NK population up to 3-fold of expression (*P*=0.001). These results suggest an NK cell expansion was the major modulation event.

### Isolated Liver NK cells from CpG-Treated Mice Attenuate HSCs Proliferation with in vitro Increased NK Adhesion

**Figure 3:**
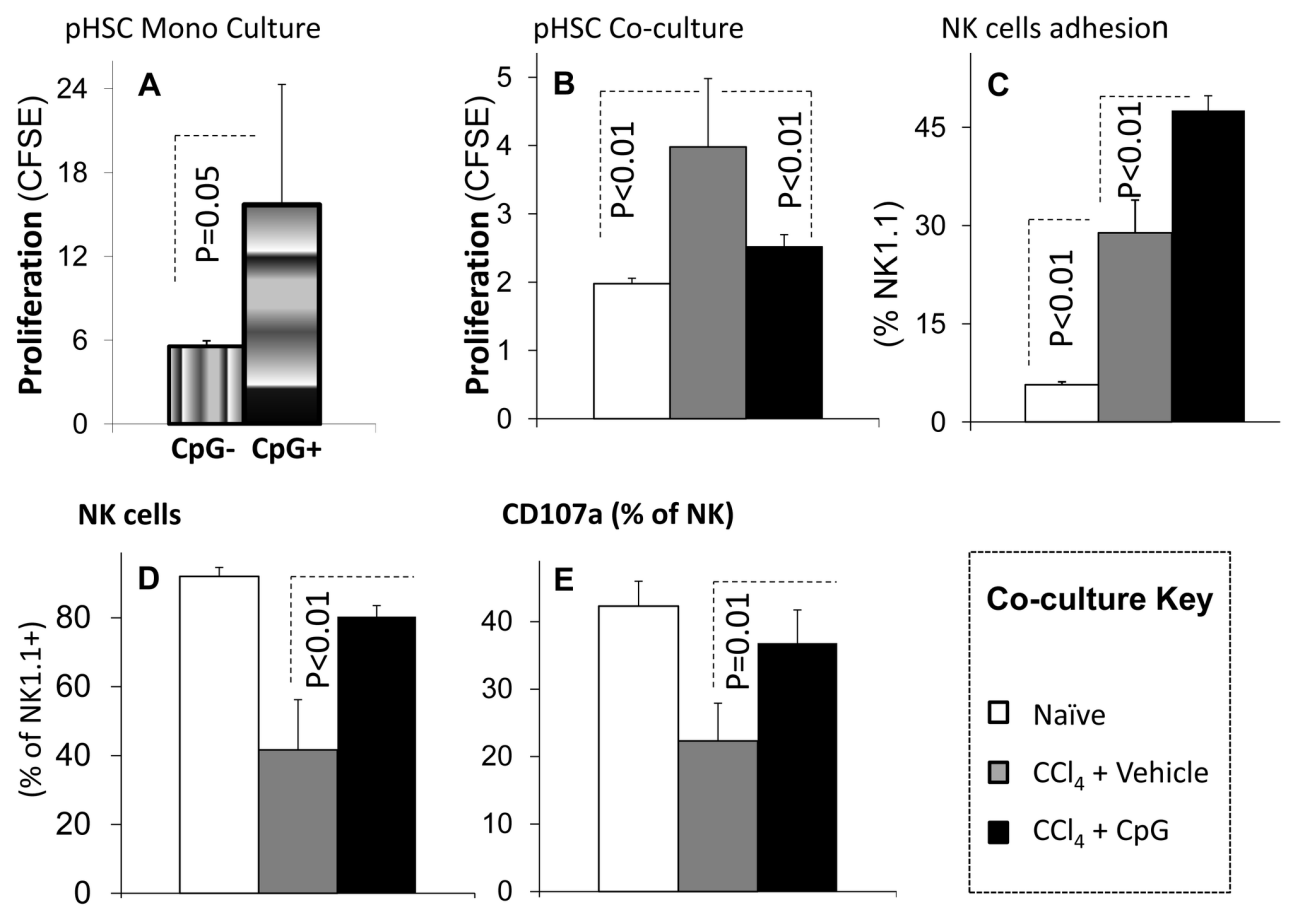
Direct versus NK-mediated in vitro effects of CpG on HSCs cultures. Liver NK cells obtained from naïve (plain bars) and fibrotic mice treated with CpG (black bars) *versus* vehicle (gray bars) were *in* *vitro* co-cultured with WT isolated HSCs; then HSCs proliferation were determined as described in Materials and Methods. A) Isolated HSCs mono-cultured with 1% FCS-enriched DMEM showed a significant increase of proliferation following direct CpG incubation (vertical gradient bars) as compared to DMEM vehicle-treated cells (horizontal gradient bars). B) Co-culture with the CpG-treated NK cells decreased HSCs proliferation (*P*<0.01) as compared to controls. C) Co-culture of liver NK cells from fibrotic CpG-treated mice with HSCs increased their adherence (*P*<0.01) as compared to the vehicle-treated fibrotic NK cells. D & E) NK increased adherence (*P*<0.01) and cytotoxic marker CD107a (*P*=0.01) when HSCs were cultured with lymphocytes from fibrotic CpG-treated mice.

Human as well as murine HSCs express TLR9 [9] and are activated by CpG [12]. Consistently, we also demonstrate herein an *in vitro* activation of quiescent primary HSCs monoculture following direct CpG incubation. Isolated HSCs showed a significantly increased proliferation following direct CpG incubation *in vitro* as compared to vehicle-treated cells (Figure 3A) suggesting that the direct pro-fibrotic effects of CpG on HSCs could not explain the in vivo CpG anti-fibrogenic results (Figure 1 & 2).

Liver NK cells obtained from fibrotic mice treated with CpG *versus* vehicle were co-cultured *in vitro* with WT isolated HSCs; then HSCs proliferation were determined after 5 days as described in Materials and Methods. Co-culture with the CpG-treated NK cells decreased HSCs proliferation (*P*<0.01) as compared to vehicle-treated control NK cells (Figure 3B). We have previously showed co-culture of lymphocytes with HSCs led to their adherence and phagocytosis [23]. Similarly, co-culture of liver NK cells from fibrotic CpG-treated mice with HSCs increased their adherence (*P*<0.01) as compared to the vehicle-treated fibrotic NK cells (Figure 3C). Specifically, NK cell adherence was increased (*P*<0.01, Figure 3D) their degranulation marker CD107a were also increased (*P*=0.01), indicating an anti-fibrotic NK cell activity (Figure 3E). 

### Increased Senescence of HSCs in TLR9^-/-^ Mice May Explain Decreased Fibrosis in Spite of Increased Liver Injury

To further explain the role of TLR9 in hepatic fibrosis, the CCl_4_ fibrosis model was induced for 4 weeks in WT and TLR9^-/-^ mice and was compared to naïve states. Serum ALT levels were assessed and isolated livers from the animal groups were evaluated for the extent of fibrosis by the Bioquant® computerized analysis of the relative fibrosis areas and by liver expressions. 

**Figure 4:**
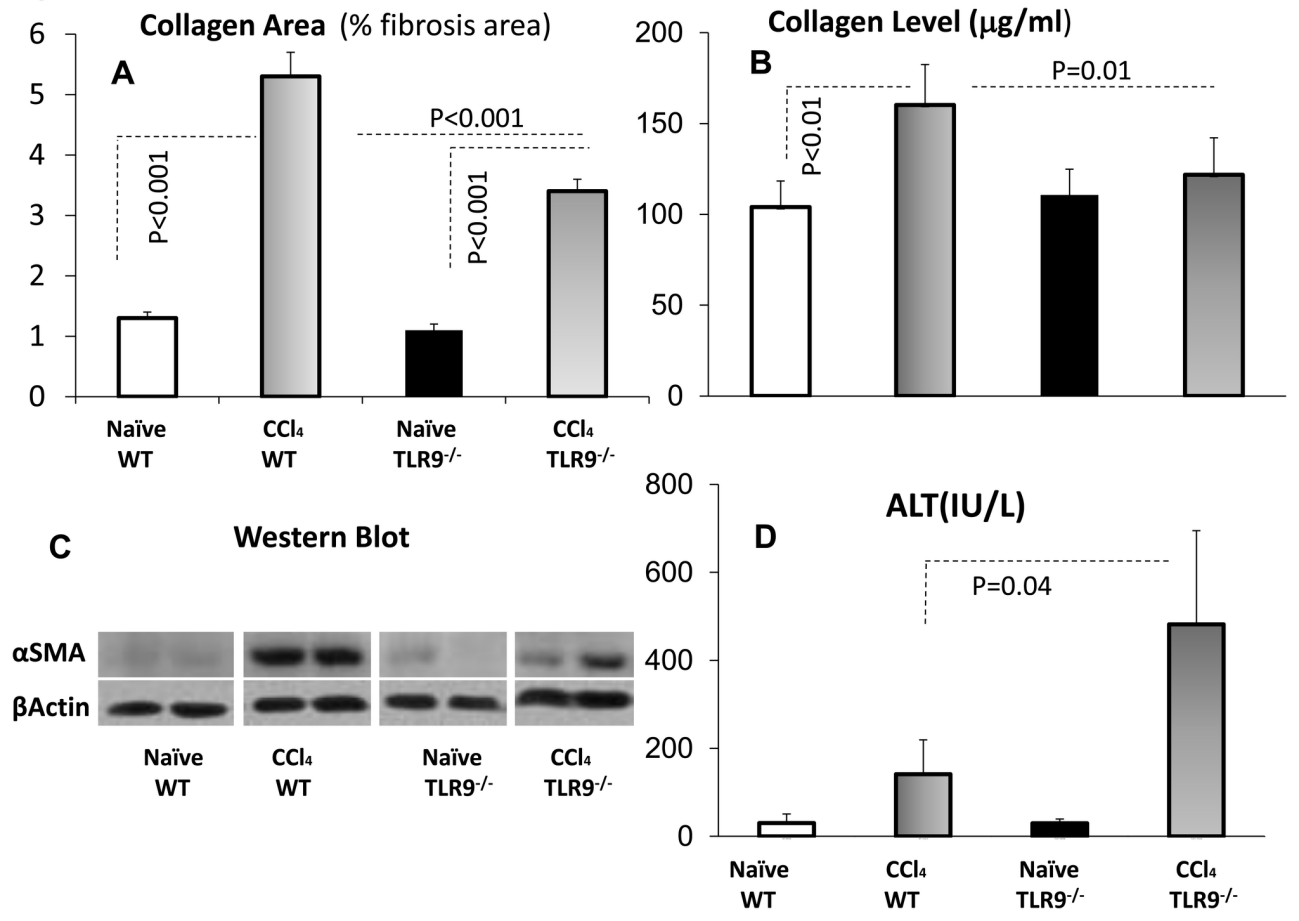
TLR9^-/-^ attenuates fibrosis but increases liver injury and senescence. The CCl_4_ fibrosis model was induced for 4 weeks in WT (horizontal gradient bars) and TLR9^-/-^ (vertical gradient bars) mice as compared to naïve states (plain and black bars, respectively). A) Collagen area following CCl_4_ induction in WT and TLR9^-/-^ animals led to an increased percent of collagen area (*P*<0.001). Hepatic collagen area in the fibrotic TLR9^-/-^ mice was significantly lower (*P*<0.001) as compared to fibrotic WT rodents. B) Collagen levels from the hepatic hydroxyproline contents showed similar patterns as the collagen area. C) Western blotting of the liver protein extracts revealed a significant reduction in αSMA protein expression as compared to WT (two representative bands for each group are shown. D) Compared to WT, serum ALT levels were significantly higher (*P*=0.04) in the fibrotic TLR9^-/-^ mice. The data represent the mean ± SD of 16 animals/group.

CCl_4_ induction in WT animals led to severe fibrosis as depicted by an increased percent of collagen area from 1.3%±0.08 in naïves to 5.3%±0.25 (*P*<0.001). The average hepatic collagen area was 1.1%±0.06 in naïve and increased to 3.39%±0.27 (*P*<0.001) in the fibrotic TLR9^-/-^ mice, which is significantly lower (*P*<0.001) compared to fibrotic WT mice (Figure 4A). Additional biochemical readout of fibrosis was performed. The hepatic hydroxyproline contents reflecting the collagen area was determined in each of the liver homogenates of each animal group. Figure 4B showed similar consistent patterns to collagen area as quantified by the Sirius red stain, which confirmed a reduction of % fibrosis area in TLR9 KO (*P=0.01*).

Liver protein extracts showed also a reduction in αSMA expression in fibrotic TLR9^-/-^ mice compared to WT fibrotic animals (Figure 4C), indicating a lower amount of activated HSCs in these mice. 

**Figure 5:**
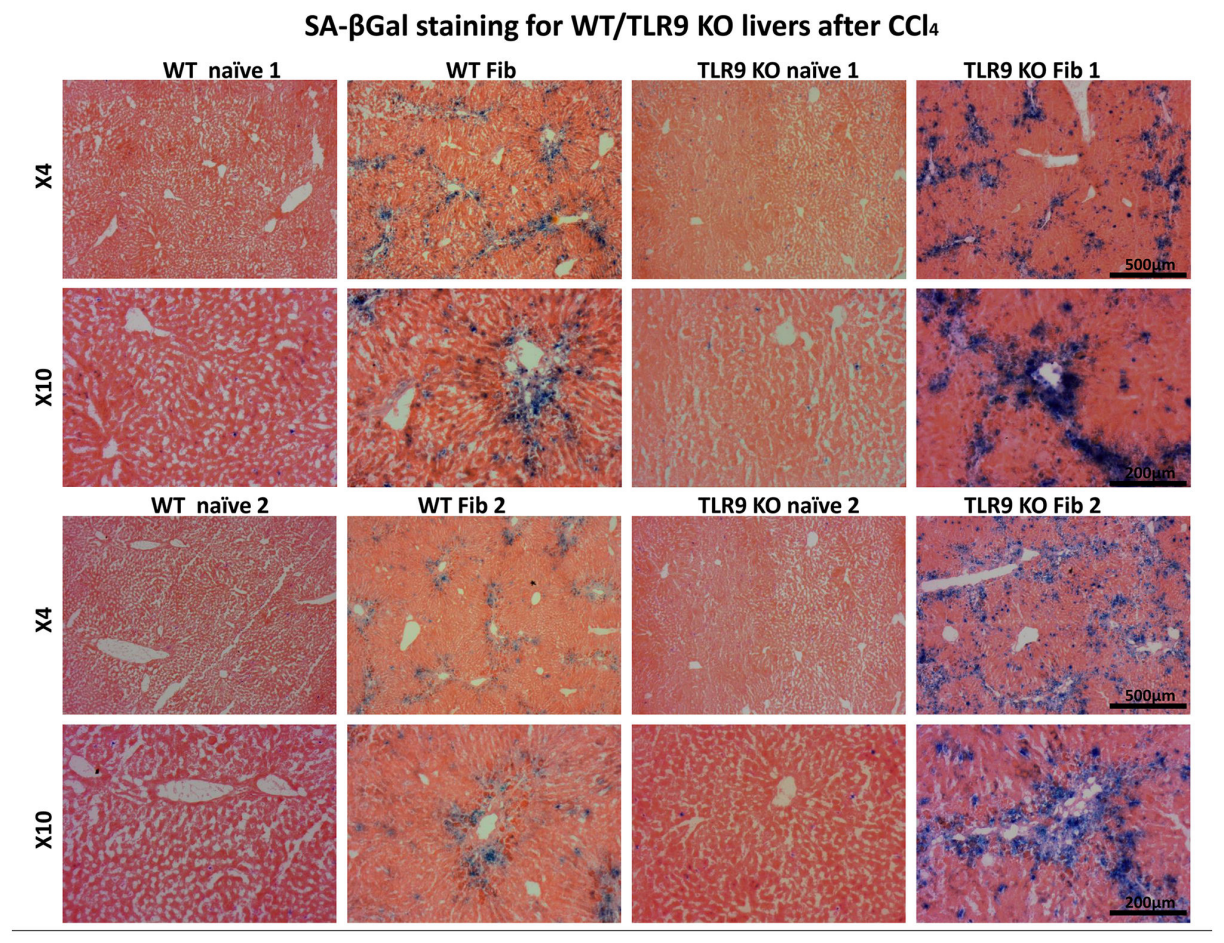
Livers of fibrotic TLR9^-/-^ have increased senescence. SA-β-gal staining of liver sections from CCl_4_ and vehicle-treated (control) in WT and TLR9^-/-^ mice reveals increase in presence of senescent cells in TLR9^-/-^ fibrotic livers. The data presented were obtained from 2 representative liver samples from different experiments showed in two magnifications (4X and 10X).

Although TLR9^-/-^ mice could not activate HSCs, their serum ALT levels were significantly higher (*P*=0.04) indicating increased liver injury (Figure 4D). Therefore, it is possible that in TLR9^-/-^ mice, HSCs become senescent shortly following activation and therefore are not able to contribute to increase in the activated HSCs population and extracellular matrix deposition in fibrotic scars. Indeed, increased levels of the senescence marker p15 INK4B were also observed in liver protein extracts by western blotting (data not shown). Senescent cells, identified by positive SA-β-gal staining, were more abundant on sections of fibrotic livers of TLR9^-/-^ mice comparing to WT fibrotic livers; however liver sections from naïve WT and TLR9^-/-^ mice showed no difference in SA-β-gal activity (Figure 5).

Yet, we need to answer whether TLR9^-/-^ lymphocytes or resident liver cells are responsible for the phenotype we observed in TLR9^-/-^ mice. The following adoptive transfer model provides answers.

### TLR9^-/-^ Lymphocytes Transferred to WT Recipients Increased Fibrosis Responses, but TLR9^-/-^ Recipients Could Not Show Sufficient Responses to Any Lymphocyte Strain

**Figure 6:**
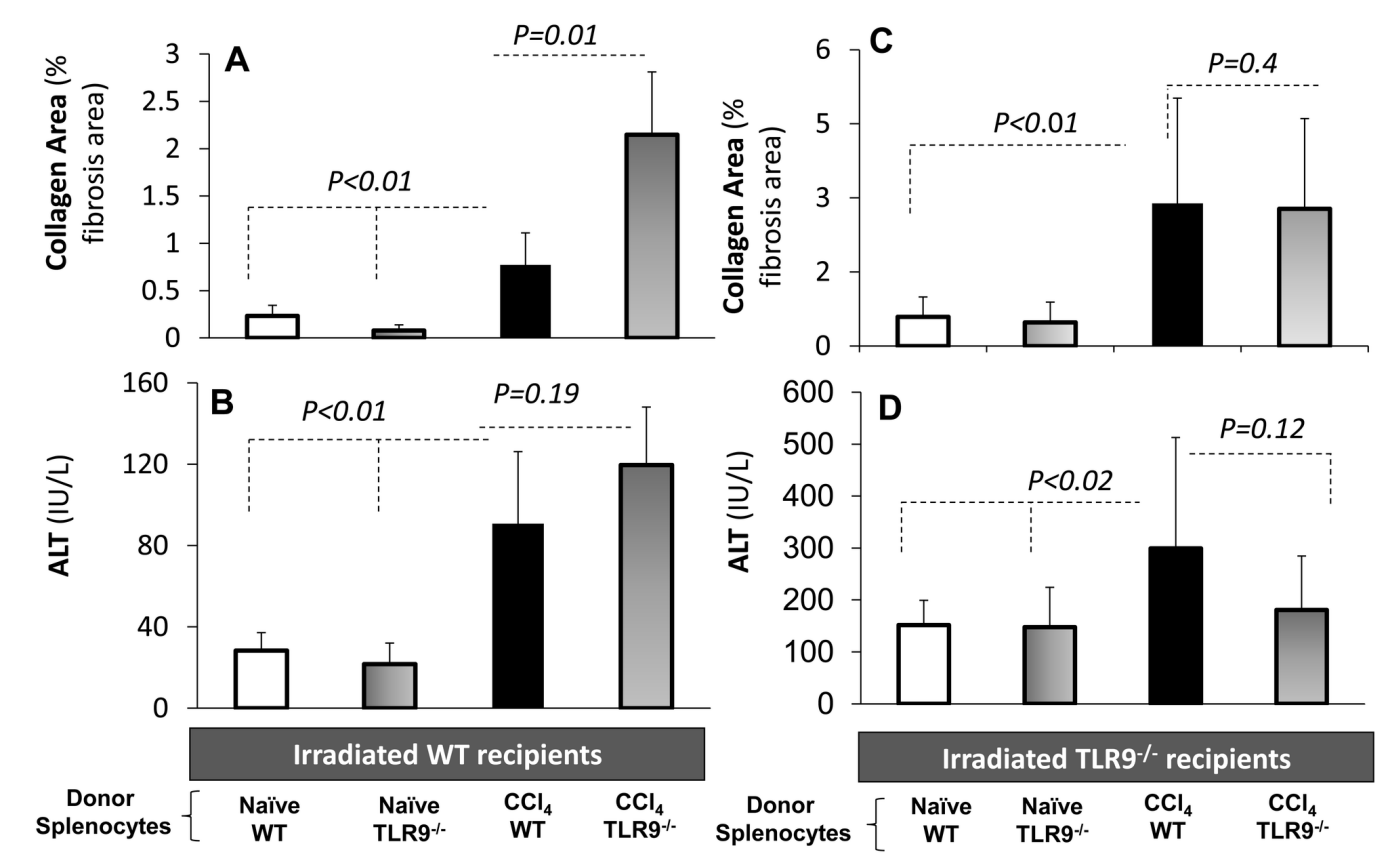
*In* *vivo* adoptive transfer model isolates lymphocyte outcome of each strain. Irradiated WT or TLR9^-/-^ recipient mice were reconstituted by naive WT or TLR9^-/-^ donor lymphocytes, along with CCl_4_ fibrosis induction, as described in Materials and Methods. Accordingly, there were 4 groups of the WT recipients and another 4 groups of TLR9^-/-^ recipients. Plain and black bars are reflecting naïve recipients reconstituted with WT or TLR9^-/-^ donor lymphocytes, respectively. Horizontal and vertical gradient bars reflect fibrotic recipients reconstituted with WT or TLR9^-/-^ donor lymphocytes, respectively. Recipient livers were assessed for histology collagen area, and serum ALT levels were measured. A) Collagen area significantly increased following CCl_4_ induction in both groups. Significant fibrosis exacerbation among WT recipients was observed when donor of lymphocytes was TLR9^-/-^ compared to WT lymphocytes (*P*<0.01). B) ALT serum levels significantly increased (*P*<0.01) following fibrosis compared to naive recipients. TLR9^-/-^ fibrotic recipients reconstituted with TLR9^-/-^ or C) WT lymphocytes achieved a significant fibrosis (*P*<0.01) and D) serum ALT (*P*<0.02) progression when compared to naives. However, both fibrotic groups were similar and showed no hepatic fibrosis or serum ALT changes.

Our results at this stage suggest that TLR9^-/-^ lymphocytes are more fibrogenic, while CpG therapy exerts an anti-fibrotic effect via WT lymphocytes, mainly through the NK cell responses. However, the lower TLR9^-/-^ fibrosis in the presence of higher liver injury suggests increase in activated HSCs senescence in TLR9^-/-^ animals. To extend and assess this hypothesis; we aimed to investigate the fibrotic functionality and characteristics of TLR9^-/-^ exclusively through lymphocytes. For this purpose, we established an adoptive transfer model of lymphocyte reconstitution from donors to irradiated recipients, WT or TLR9^-/-^ strains. Irradiated WT or TLR9^-/-^ recipient mice were reconstituted by naive WT or TLR9^-/-^ donor lymphocytes once per week for 4 weeks via intra-peritoneal injections, along with CCl_4_ fibrosis induction, as described in Materials & Methods. Accordingly, there were 4 groups of the WT recipients and another 4 groups of the TLR9^-/-^ recipients. In each recipient strain, naive *versus* fibrosis was correlated in the presence of WT *versus* TLR9^-/-^ lymphocyte reconstitutions (Figure 6). In all animals of 8 groups, recipient livers were assessed for fibrosis by αSMA liver protein expression and liver histology of collagen area, and serum ALT levels were measured.

A significant fibrosis exacerbation among WT recipients was observed when donor of lymphocytes was TLR9^-/-^ compared to WT lymphocytes. Fibrosis progression was quantified by a significant increase in the Sirius red fibrosis area (*P*<0.01, Figure 6A) and confirmed by similar pattern of hepatic αSMA protein expressions. When compared to naive recipients, ALT serum levels significantly increased (*P*< 0.01) following fibrosis, but were not much affected by the TLR9 lymphocyte background (Figure 6B). These results suggest that TLR9^-/-^ lymphocytes have a pro-fibrogenic impact on liver pathology. This result is in line with the opposed CpG immune fibrotic outcome in WT animals (Figure 1). 

TLR9^-/-^ fibrotic recipients reconstituted with TLR9^-/-^ or WT lymphocytes also achieved a significant fibrosis progression when compared to naïves. However, both fibrotic groups were similar and showed no changes in the severity of hepatic fibrosis (*P*=0.4, Figure 6C), ALT serum levels (*P*=0.12, Figure 6D) or αSMA protein expressions. 

## Discussion

The findings presented in the current study uncover opposed TLR9 roles to modulate hepatic fibrosis. There is now ample evidence indicating that liver inflammation and fibrosis are mediated by the innate immune response, which is regulated among others by Toll-like receptors [28]. TLR9 pathways are involved in the modulation of liver diseases [29]. Disruption of TLR9, MyD88, or IL-1β signaling significantly attenuates liver fibrosis [30]. TLR ligands can directly target HSCs via binding to TLR9, followed by inhibition of hepatic stellate cell chemotaxis and induction of their activation, ultimately leading to liver fibrosis [28]. The discrepancy among these studies raised requirements for clarification. TLR9 activation by CpG motifs was reported to activate isolated HSCs *in vitro* [9]. Moreover, attenuated hepatic fibrosis in TLR9^-/-^ animal models supported the pro-fibrogenic effect of TLR9 activation [9,31]. A direct TLR9 activation of HSCs by apoptotic hepatocyte DNA was accordingly proposed to induce a pro-fibrotic effect since apoptotic hepatocytes activate fibrosis [28]. This pro-fibrogenic effect was therefore a result of a direct HSCs activation by a TLR9 agonist such as CpG, regardless of immune involvement. While CD8 cells are directly mediating fibrosis [23], NK cells provide an anti-fibrotic role [14,16], suggesting those responses to be TLR9-mediated as well. Particularly, CpG increases NK responses in different models [32], supporting a potential anti-fibrotic effect. 

In the current study with a CCl_4_-induced fibrosis model, treatment with CpG attenuates the WT hepatic fibrosis, serum ALT levels (Figure 1C) and liver CD8 infiltrates (Figure 2). In addition, CpG augments serum IL-10 and IFNγ levels together with a marked increase of liver NK cells. These results indicate an anti-fibrotic (IFNγ and IL-10), pro-inflammatory (IFNγ) as well as anti-inflammatory (IL-10) modulations in the CCl_4_ fibrotic groups receiving the CpG motifs. TLR9 targets multiple cell types to promote mixed inflammatory responses following TLR9 activation. The TLR9 activation on Kupffer cells leading to inflammation and activation of TLR9 on T cells [30], NK T cells, neutrophils, and sinusoidal endothelial cells also results in the secretion of pro-inflammatory cytokines in various liver inflammation models [32,33] . By contrast, TLR9 activation on conventional dendritic cells results in IL-10 secretion that subsequently attenuates the inflammatory response and liver ischemia injury [32]. The TLR9 activation by agonists was also reported to affect innate and adaptive immunity, leading to the differentiation of TH1 cells and increased CTLs, increased secretion of IFNγ and IL-10 cytokines [34]. CpG increases HCV-specific IFNγ CD4+ as well as CD8+ producing T cell responses [35,36] suggesting an anti-fibrotic effect.

 A direct CpG effect under monoculture conditions [28] is reproduced here-in (Figure 3A), and is mimicking the apoptotic hepatocyte DNA. Both induce the differentiation of mouse and human HSCs via TLR9 and inhibit platelet-derived growth factor mediated HSCs chemotaxis [22]. On the other hand, the current anti-fibrotic CpG outcome from *in vivo* WT results from the CCl_4_ model (Figure 1) is not in line with the direct *in vitro* HSCs activation. An opposed indirect CpG effect was found via lymphocyte alterations. Isolated liver NK cells from fibrotic mice treated with CpG *versus* vehicle were co-cultured *in vitro* with naive primary isolated WT HSCs. Indeed, liver NK cells obtained from CpG-treated mice were significantly less potent in inducing proliferation of HSCs, as a marker for HSCs activation (Figure 3B). The adhered HSCs/NK cells increased (Figure 3C, 3D) in the case of CpG-treated lymphocytes and were significantly more cytotoxic according to CD107a, a marker of degranulation (Figure 3E). The CpG anti-fibrotic effect was therefore mediated by the immune system, including the adoptive T cell response, albeit mainly the NK innate immunity. Together with its direct increase of HSCs activation and indirect HSCs alleviation via the lymphocytes, CpG stimulation demonstrates therefore, an evidence of dual effects under hepatic fibrosis conditions. Therefore, the CpG fibrotic outcome is a matter of balance between both pro and anti-fibrotic effects. The anti-fibrotic NK cells were augmented by CpG stimulation, along with increased NK cytotoxicity and increased adhesion to HSCs. Adhered NK-cells were reported to exert anti-fibrotic effects via direct interactions with HSCs [12,14,19,37]. A dual role in inflammation and NK-cell responses was also reported after TLR9 triggering in mouse models of microbial infection [38]. The discrepancy demonstrated in the TLR9^-/-^ fibrosis model (attenuated fibrosis with higher ALT and senescence) further enforces dual effects. Increased senescence of activated HSCs in TLR9^-/-^ livers provides a fair explanation for the lack of fibrosis induction in-spite of increased liver injury (Figure 4). Furthermore, increased senescence in TLR9^-/-^ livers may explain the reported failure of isolated HSCs to induce MCP-1 following CpG stimulation [28].

 Accordingly, a couple of challenging questions may arise from the TLR9^-/-^ fibrosis model: Are TLR9^-/-^ lymphocytes more fibrogenic? And why the fibrosis outcome is attenuated even with fibrotic lymphocytes? To address these points, we first reproduced the reported attenuation of hepatic fibrosis in TLR9^-/-^ rodents as compared with WT (Figure 4A). However, elevated serum ALT levels in the TLR9^-/-^ fibrotic group (Figure 4B) suggest an increase of hepatoctye apoptosis or of pro-fibrogenic lymphocytes. In both scenarios, hepatocyte injury could not develop fibrosis response due to increased senescence of activated HSCs in TLR9^-/-^ mice. To confirm our hypothesis, we planned the adoptive transfer model, described in Materials and Methods, that enables the isolation of lymphocyte effects. A significant fibrosis exacerbation among WT recipients was observed when donor of lymphocytes was TLR9^-/-^ compared to WT lymphocytes. On the other hand, TLR9^-/-^ fibrotic recipients reconstituted with TLR9^-/-^ or WT lymphocytes showed no changes in severity of hepatic fibrosis or serum ALT levels. These results suggest that the unchanged fibrosis phenotype following the CCl_4_-treated TLR9^-/-^ mice, whether reconstituted with the WT or the TLR9^-/-^ lymphocytes, is actually a result of response-inability due to early senescence of TLR9^-/-^ activated HSCs. These results indicate and emphasize that the existence of the receptor on the lymphocytes is necessary to inhibit fibrosis and decrease inflammation.

 In summary, the CpG fibrosis outcome is the balance between anti-fibrotic immune cells and direct pro-fibrotic HSCs activation. TLR9- activation had inconsistent effects on lymphocytes and HSCs. The net balance of TLR9 activation in WT, displayed significant anti-fibrotic activity, accompanied by CD8 suppression and increased NK-cells, activity and adherence to HSCs. On the other hand, TLR9^-/-^ lymphocytes were pro-fibrotic and pro-inflammatory causing an increase in the αSMA expression and ALT serum levels when transferred to the WT recipient. However, TLR9^-/-^ recipients did not respond to the activation stimuli, suggesting early senescence of activated HSCs. TLR9 appears to have a dual impact on fibrosis outcome, on lymphocytes as well as on the HSCs.
